# Supplementary material for: Dual lysine and N‐terminal acetyltransferases reveal the complexity underpinning protein acetylation
Source: Mol Syst Biol. 2020 Jul 7;16(7):e9464. doi: 10.15252/msb.20209464 (PMC7339202; doi:10.15252/msb.20209464)
Supplement: Supplementary file 1 — Expanded View Figures PDF [file MSB-16-e9464-s001.pdf]

Expanded View Figures

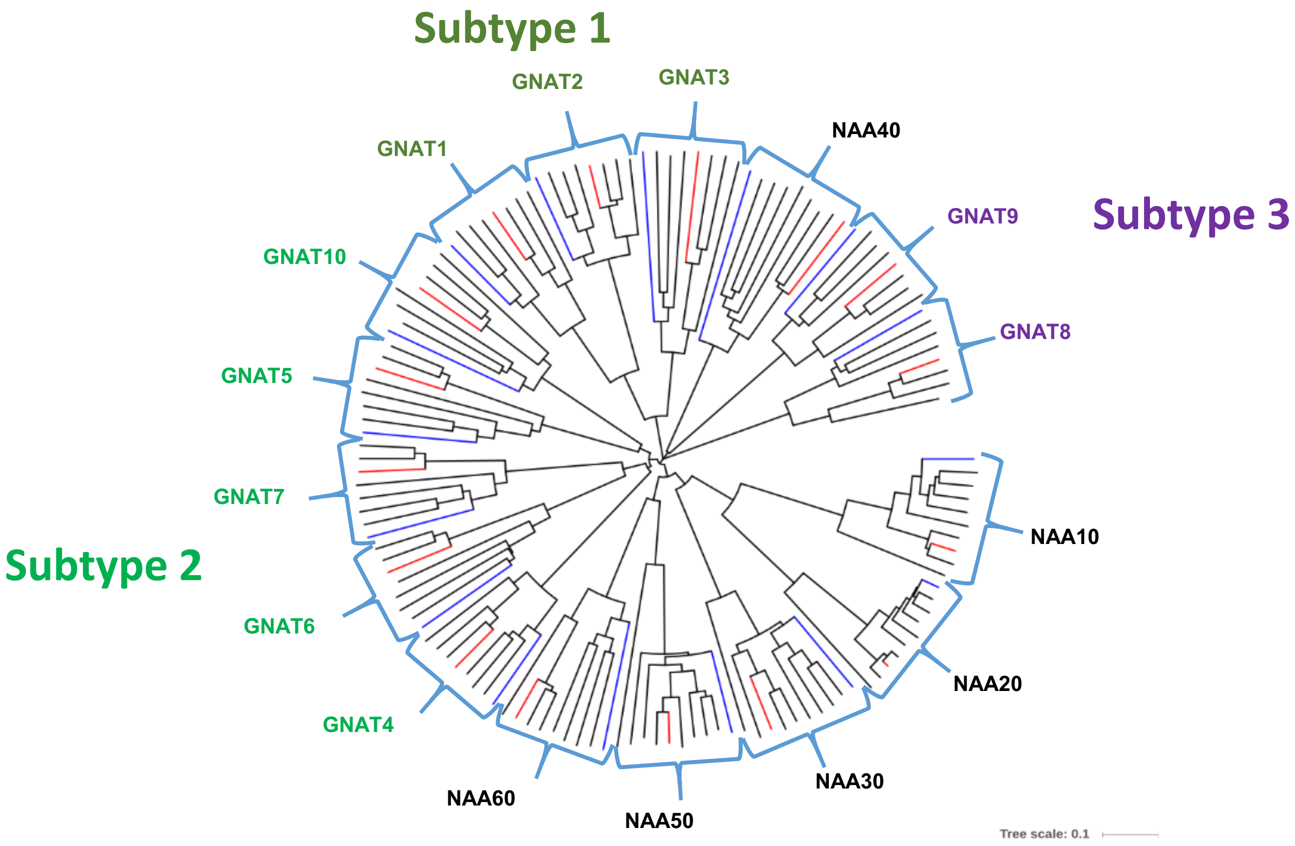

Figure EV1. Phylogenetic tree of NAT/KATs based on protein sequence comparison.

ClustalW alignment was performed using the homologous protein sequence of NAA10, NAA20, NAA30, NAA40 (except for *C. reinhardtii*), NAA50, NAA60, GNAT1 (At1g26220), GNAT2 (At1g32070, NSI (Koskela *et al*, 2018)), GNAT3 (At4g19985), GNAT4 (At2g39000, AtNAA70 (Dinh *et al*, 2015)), GNAT5 (At1g24040), GNAT6 (At2g06025), GNAT7 (At4g28030), GNAT8 (At2g39020), GNAT9 (At2g04845), and GNAT10 (At1g72030) from *Arabidopsis thaliana*, *Medicago truncatula*, *Vitis vinifera*, *Populus trichocarpa*, *Zea mays*, *Musa acuminata*, *Triticum aestivum*, *Solanum lycopersicum*, *Chlamydomonas reinhardtii*, *Oryza sativa*, *Marchantia polymorpha* and displayed in a circular mode using the iTOL tool (https://itol.embl.de). Plastid-associated GNATs are colored in green, while the other two GNATs are shown in purple.

 © 2020 The Authors

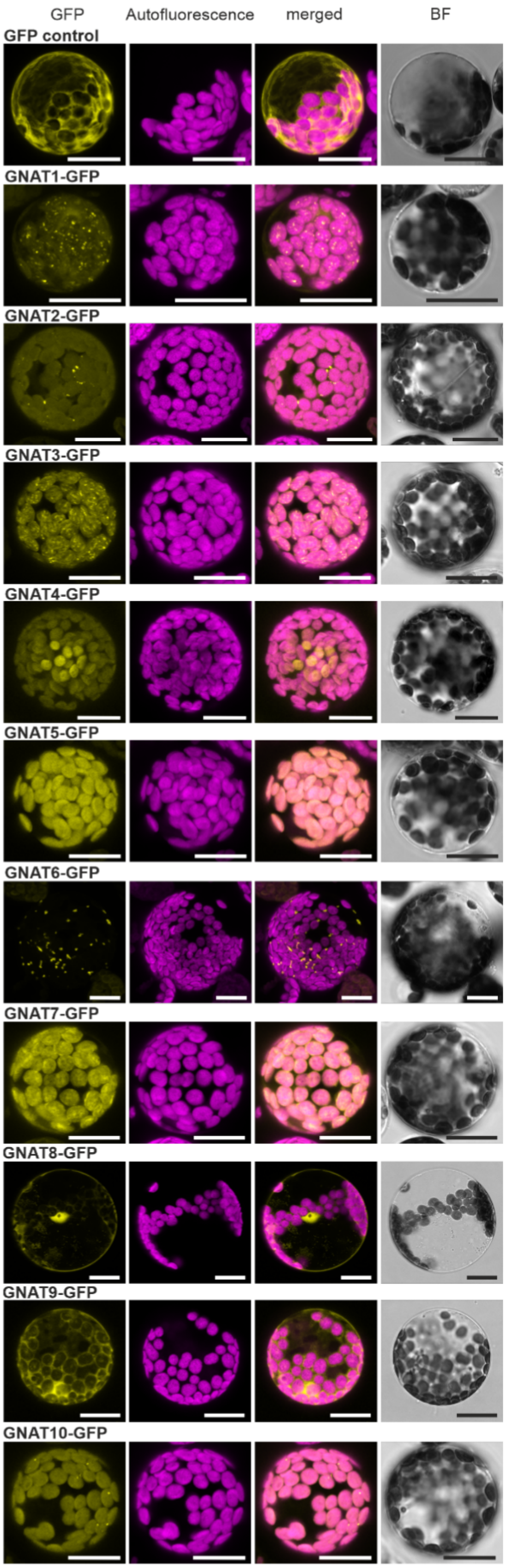

Figure EV2. Subcellular localizations of *Arabidopsis* GNAT proteins.

Confocal microscopy image of *Arabidopsis* protoplasts expressing GNAT-GFP (35S:*GNAT-GFP*) protein constructs or GFP alone (35S:*GFP*). *Arabidopsis* protoplasts were either transiently transformed (GNAT1, 2, 3, 4, 5, 6, 7, 10) or prepared from stable, GNAT overexpressing plant lines (GNAT8, 9). GFP reporter signal (yellow), chlorophyll autofluorescence (pink), merged fluorescence signals, and the bright field channel (BF). The scale bar represents a size of 20 μm.

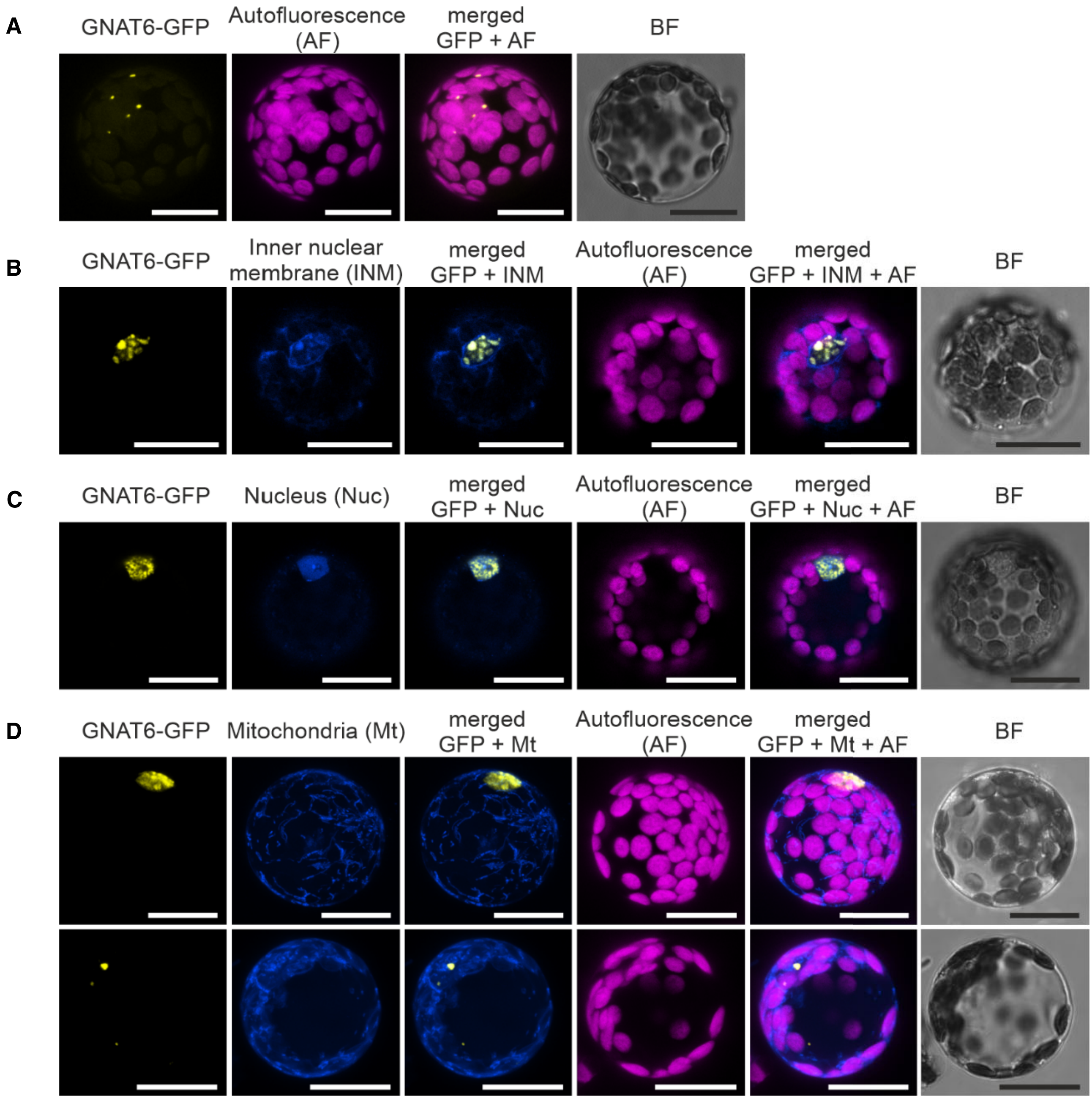

Figure EV3. Co-expression of GNAT6-GFP with subcellular localization markers.

A–D Confocal laser scanning microscopy images of *Arabidopsis* Col-0 protoplasts transiently expressing a GNAT6-GFP (35S:*GNAT6-GFP*) fusion protein. The GNAT6-GFP signal shows a spotted pattern in different subcellular compartments. When indicated, protoplasts were either (B) transiently co-transformed with a plasmid enabling the expression of an inner nuclear membrane marker (INM: SUN1-OFP, Rips *et al*, 2017), (C) treated with Hoechst 33342 (Thermo Fisher) reagent for DNA staining for identification of the nucleus (Nuc), or (D) treated with MitoTracker (Mt: MitoTracker Orange CMTMRos, Invitrogen) for staining of mitochondria. GFP reporter signal (yellow), chlorophyll autofluorescence (pink), merged fluorescence signals and the bright field channel (BF). The scale bar represents a size of 20 μm.

 © 2020 The Authors

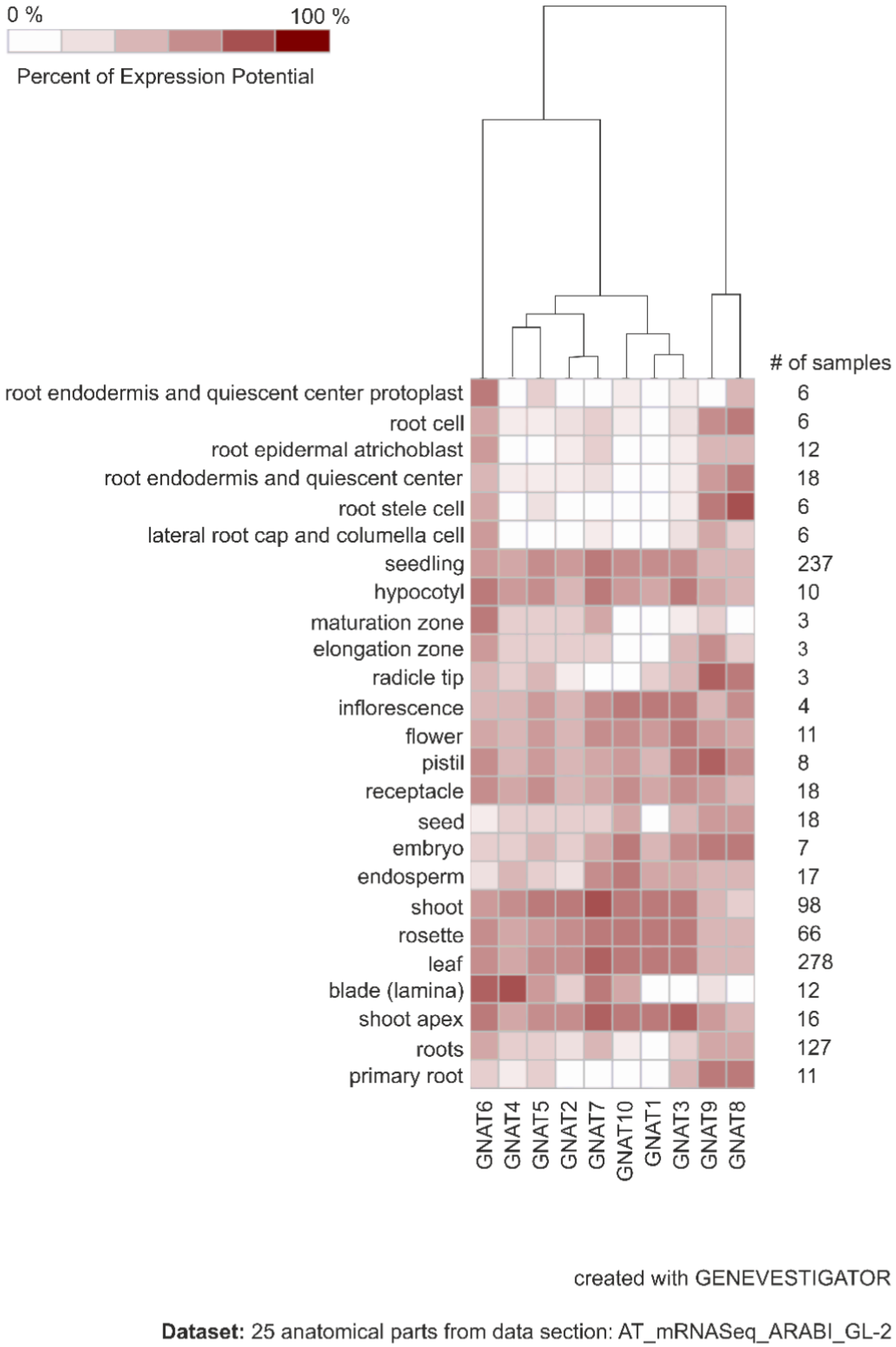

Figure EV4. Hierarchically clustered heatmap of tissue-specific expression of genes encoding the GNAT proteins.
Tissue-specific gene expression data (RNAseq) were obtained from GENEVESTIGATOR (Hruz *et al*, 2008).

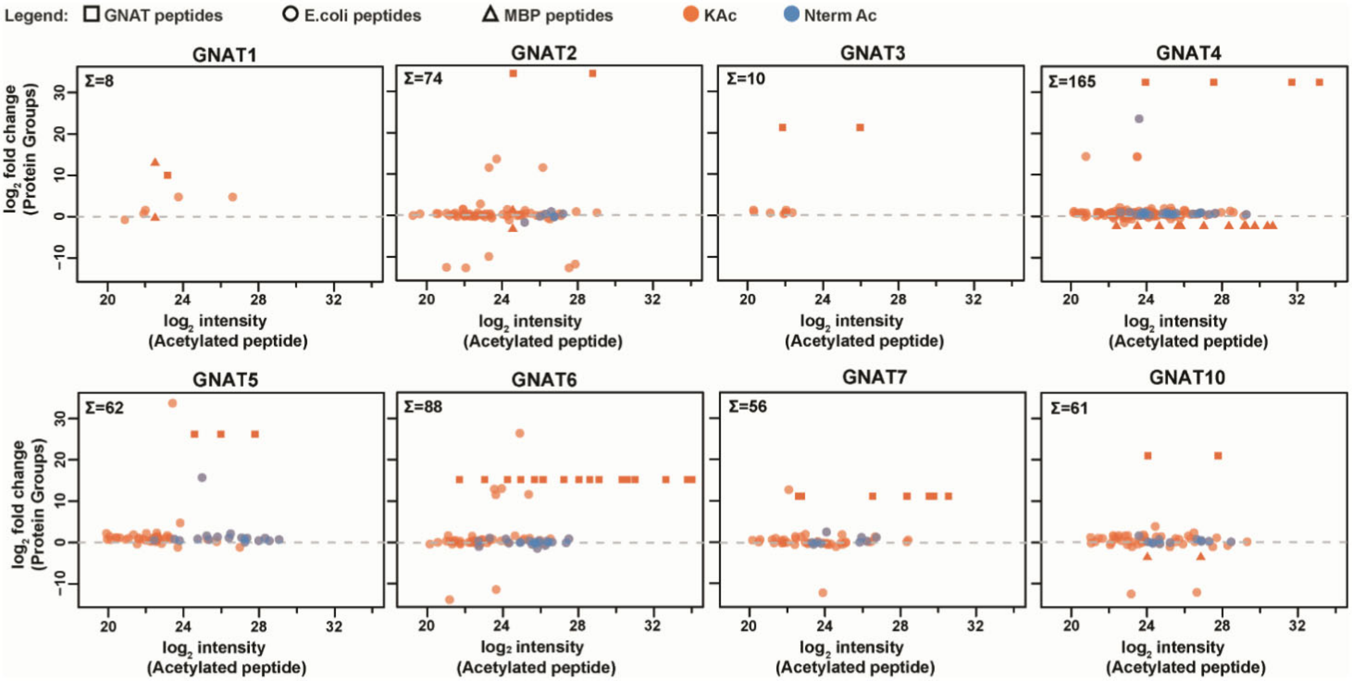

Figure EV5. Correlation between protein abundance and acetylation level on newly acetylated proteins after GNAT-MBP expression.

Average values from two biological replicates (with label swap) are plotted. Number on the left upper corner indicates newly acetylated proteins where peptide and protein abundance were quantified. Protein abundance is plotted as $\log_2$ ratio from cells with GNAT-MBP and MBP expression. Acetylated peptides from to the respective GNAT proteins are indicated as a box, acetylated peptides from the MBP fusion protein are indicated as a triangle, acetylated peptides from *Escherichia coli* are indicated as a circle, KA peptides are highlighted in orange, and NTA peptides are highlighted in blue.

 © 2020 The Authors

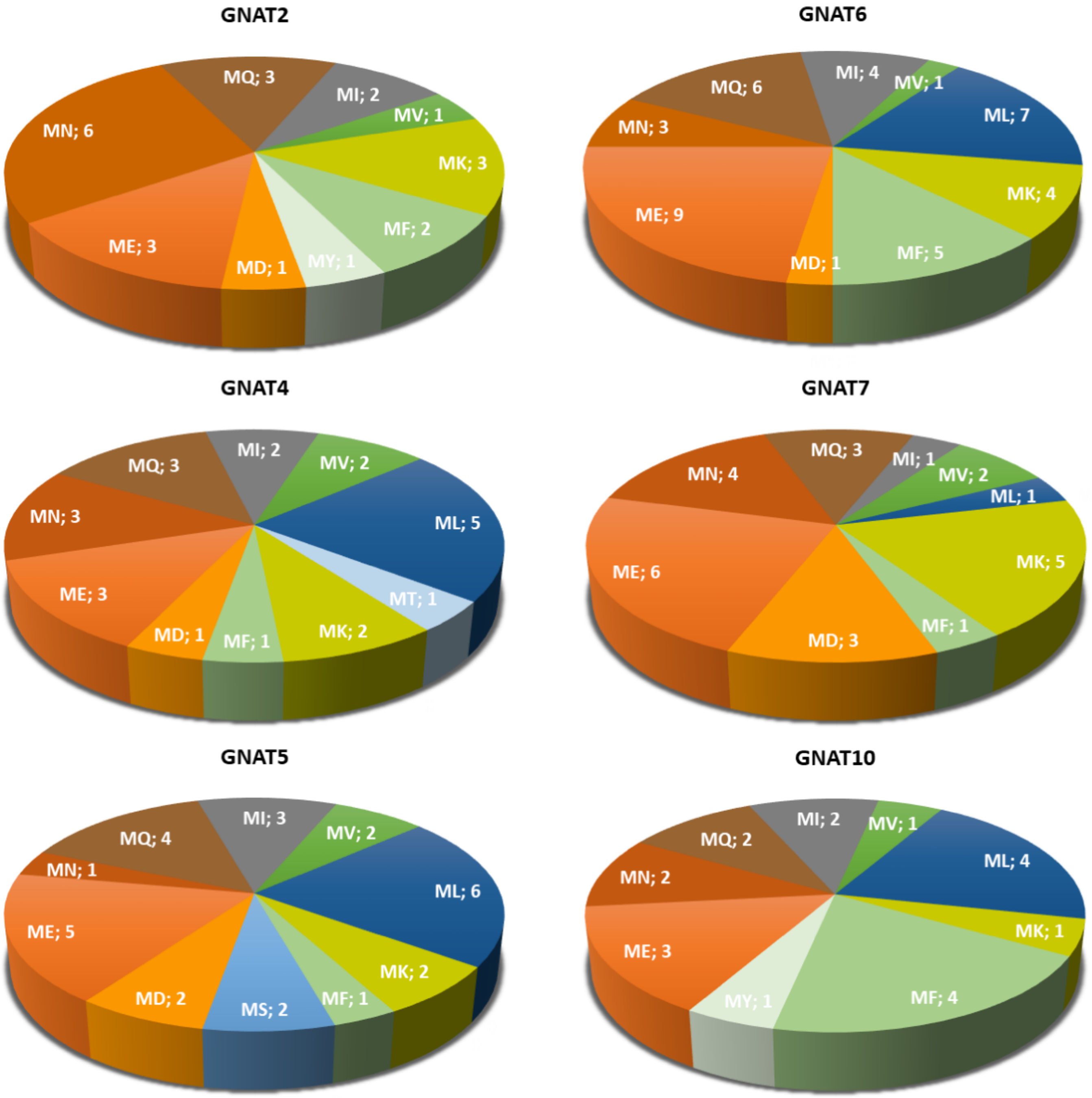

Figure EV6. Specificity of GNATs on Met-starting protein substrates.

The set of substrates starting with Met colored in light and dark orange in Fig 4 is analyzed according to the nature of the following residue (X). The same color code for each MX subset is used in each pie chart.
