# Supplementary material for: Dual lysine and N‐terminal acetyltransferases reveal the complexity underpinning protein acetylation
Source: Mol Syst Biol. 2020 Jul 7;16(7):e9464. doi: 10.15252/msb.20209464 (PMC7339202; doi:10.15252/msb.20209464)
Supplement: Supplementary file 2 — Table EV1 [file MSB-16-e9464-s002.docx]

**Table EV1. Characteristics of candidate GNAT proteins from Arabidopsis.** Subcellular localization was predicted with Target P-1.1. However, only nine of them possess a clear TP, of which the length was determined using different prediction tools (i.e., N-TerPred, ChloroP, TargetP, Suba, and multiple sequence alignment of plant orthologues). TP = transit peptide, pI = isoelectric point, w/o = without, aa = amino acid.

| Name | Accession  Number | Target Prediction  (TargetP-1.1) | No.  of  aas | Mol. Mass  [kDa] | Length of TP [No. of aas] | Mol. Mass  w/o TP [kDa] | pI  w/o TP |
| --- | --- | --- | --- | --- | --- | --- | --- |
| GNAT1 | AT1G26220.1 | Plastid | 197 | 21.9 | 46 | 17.0 | 7.73 |
| GNAT2 | AT1G32070.2 | Plastid | 258 | 28.6 | 40 | 24.3 | 4.76 |
| GNAT3 | AT4G19985.1 | Mitochondrion | 237 | 26.5 | 41 | 22.1 | 5.44 |
| GNAT4 | AT2G39000.1 | Plastid | 291 | 32.2 | 61 | 25.6 | 9.11 |
| GNAT5 | AT1G24040.1 | Plastid | 319 | 36.0 | 55 | 30.0 | 5.53 |
| GNAT6 | AT2G06025.1 | Mitochondrion | 288 | 33.6 | 45 | 28.2 | 8.44 |
| GNAT7 | AT4G28030.1 | Plastid | 274 | 30.4 | 38 | 26.4 | 5.89 |
| GNAT8 | AT2G39020.1 | Plastid | 236 | 26.4 | 33 | 23.1 | 5.55 |
| GNAT9 | AT2G04845.1 | Mitochondrion | 218 | 24.5 | 0 | 24.5 | 5.31 |
| GNAT10 | AT1G72030.1 | Plastid | 256 | 28.9 | 55 | 22.9 | 6.22 |
