# Supplementary material for: Dual lysine and N‐terminal acetyltransferases reveal the complexity underpinning protein acetylation
Source: Mol Syst Biol. 2020 Jul 7;16(7):e9464. doi: 10.15252/msb.20209464 (PMC7339202; doi:10.15252/msb.20209464)
Supplement: Supplementary file 3 — Table EV2 [file MSB-16-e9464-s003.docx]

**Table EV2. The consensus Ac-CoA binding domain in Arabidopsis GNATs.**

The main and secondary Ac-CoA binding motifs found in the different Arabidopsis GNATs are reported. The consensus pattern derived from plant orthologues is provided, confirming the extent of the divergence at a larger scale. The usual Ac-CoA-BD pattern is associated to position 1, 4, 6 and 9; bold letters with dark grey background are in agreement with the expected pattern, whereas italicized letters or no background are divergent. A newer conserved position appears at position 5 and 10 in light grey.

|  |  | **Ac-CoA binding domain (residues position)** | | | | | | | | | |  |
| --- | --- | --- | --- | --- | --- | --- | --- | --- | --- | --- | --- | --- |
| **GNAT** | **AcCoA-BD status** | **1** | **2** | **3** | **4** | **5** | **6** | **7** | **8** | **9** | **10** | **Orthologs consensus pattern** |
| **1** | **main** | **Q** | **S** | **C** | **G** | **L** | **G** | **K** | **A** | **V** | **M** | **QXXGLG[KR]A[VIL][MIV]** |
| **1** | **second** | **R** | **A** | **T** | **G** | ***D*** | **G** | **V** | **F** | ***N*** | ***A*** | **RAXGDX[VI]FNA** |
| **2** | **main** | **Q** | **G** | **Q** | **G** | **L** | **G** | **K** | **A** | **L** | **V** | **[QH]GQGLG[KR]X[LM][VIM]** |
| **2** | **second** | **K** | **L** | **I** | **G** | **M** | **A** | **R** | **A** | **T** | **S** | **[KRG]LIG[ML]ARATS** |
| **3** | **main** | **Q** | **R** | **M** | **G** | **I** | **G** | **K** | **L** | **I** | **V** | **[RQ]XXG[IV]GXXI[VL]** |
| **3** | **second** | **Q** | **L** | **V** | **G** | **F** | **G** | **R** | **A** | ***Y*** | ***S*** | **X[LF][IV]GFGRAXS** |
| **4** | **main** | **R** | **R** | **K** | **G** | **I** | **A** | **K** | **R** | **L** | **I** | **RXXGI[AG]KXL[IV]** |
| **4** | **second** | **R** | **R** | **T** | **G** | **I** | **A** | **Y** | **V** | ***S*** | ***N*** | **[RK]RXG[IV]AY[IV]XN** |
| **5** | **main** | **R** | **R** | **R** | **G** | **I** | **G** | **W** | **H** | **L** | **L** | **RR[RK]G[IL]GWXLL** |
| **6** | **main** | **R** | **R** | **Q** | **G** | **I** | **A** | **C** | **N** | **M** | **L** | **RR[QR]G[IV]AXXM[LMI]** |
| **7** | **main** | ***H*** | **R** | **N** | **G** | **V** | **G** | **Y** | **K** | **L** | **I** | **[HQ][RK]XG[LVFI][GA]XX[LIV][ILV]** |
| **7** | **second** | ***K*** | **P** | **E** | **G** | **I** | **G** | **V** | **D** | **F** | ***A*** | **[KR]PXXXXXXX** |
| **8** | **main** | **R** | **R** | **K** | **G** | **F** | **G** | **S** | **M** | **L** | **L** | **RXXG[FML]GXX[LM]X** |
| **8** | **second** | **R** | **L** | **T** | **G** | **D** | **A** | **L** | **E** | **A** | **F** | **R[LI]XGXALXX[YF]** |
| **9** | **main** | **R** | **G** | **K** | **G** | **L** | **G** | **K** | **E** | ***S*** | ***V*** | **[RH]XKG[LIF][GA]XX[SA][VIA]** |
| **10** | **main** | **R** | **R** | **K** | ***K*** | **M** | **A** | **S** | **T** | **L** | **L** | **R[RK]XK[VIL][AG][TS]XL[LM]** |
| **10** | **second** | ***Y*** | **V** | **S** | **G** | **L** | **A** | **V** | **S** | ***K*** | ***S*** | **[YF][VIM][ST]G[ILM][AG]VXXX** |
