# Supplementary material for: Dual lysine and N‐terminal acetyltransferases reveal the complexity underpinning protein acetylation
Source: Mol Syst Biol. 2020 Jul 7;16(7):e9464. doi: 10.15252/msb.20209464 (PMC7339202; doi:10.15252/msb.20209464)
Supplement: Supplementary file 4 — Table EV3 [file MSB-16-e9464-s004.docx]

**Table EV3. List of oligonucleotide primers used for GFP fusion and protoplast transformation**

| Description | Sequence |
| --- | --- |
| GNAT1_forward-1 | 5’-TATACCCGGGATGTTTCTCGGAGG-3’ |
| GNAT1_reverse-1 | 5’-TATAGGATCCTTTCTTGTTTCTCTGTTTGC-3’ |
| GNAT2_forward-1 | 5’-TATACCCGGGATGCTACTAATCCCA-3’ |
| GNAT2_reverse-1 | 5’-TATAGGATCCCTTTGGGTACCAAAACATG-3’ |
| GNAT3_forward-1 | 5’-CACCCTTAAGGAAATGGGTTTG-3’ |
| GNAT3_reverse-1 | 5’-TGCCTCCAAGCTCTTTGTGA-3’ |
| GNAT4_forward-1 | 5’-TATAGGATCCATGCGGAGCACAC-3’ |
| GNAT4_reverse-1 | 5’-TATAGGATCCCCGAAACTGTTCAAGAGCT-3’ |
| GNAT5_forward-1 | 5’-TATACCCGGGATGGCGGCTTTAAG-3’ |
| GNAT5_reverse-1 | 5’-TATAGGATCCCACATTTGCAGAGGAGG-3’ |
| GNAT6_forward-1 | 5’-TATACCCGGGATGTCGACGATTTC-3’ |
| GNAT6_reverse-1 | 5’-TATAGGATCCGCTTGTGTACTGGAGCAAG-3’ |
| GNAT7_forward-1 | 5’-TATACCCGGGATGGCGTTTCTCTG-3’ |
| GNAT7_reverse-1 | 5’-TATAGGATCCCATGGACATGATTGG-3’ |
| GNAT8_forward-1 | 5’-CACCGTACCAAAAAAAAATGGCA-3’ |
| GNAT8_reverse-1 | 5’-GATGTTGACCTGATCAAAAGCTTC-3’ |
| GNAT9_forward-1 | 5’-CACCAGAAGAATGGTGACAAG-3’ |
| GNAT9_reverse-1 | 5’-TGCAGTAGCTTCTCCTGATAGC-3’ |
| GNAT10_forward-1 | 5’-TATACCCGGGATGGGTCACTTGC-3’ |
| GNAT10_reverse-1 | 5’-TATAGGATCCAGAAAAGCGTTTACTCATAAGAACG-3’ |
