# Supplementary material for: Dual lysine and N‐terminal acetyltransferases reveal the complexity underpinning protein acetylation
Source: Mol Syst Biol. 2020 Jul 7;16(7):e9464. doi: 10.15252/msb.20209464 (PMC7339202; doi:10.15252/msb.20209464)
Supplement: Supplementary file 5 — Table EV4 [file MSB-16-e9464-s005.docx]

**Table EV4. List of oligonucleotide primers used for heterologous overexpression in *E. coli***

| Description | Sequence |
| --- | --- |
| GNAT1_forward-2 | 5’-GGACCATGGACTCAATCAGCGACGAAGATC-3’ |
| GNAT1_reverse-2 | 5’-GCTGGTACCTTATTTCTTGTTTCTCTGTTTGCG-3’ |
| GNAT2_forward-2 | 5’-CTGCCATGGCGAGTAGAAAGTTGAAGACTTTG-3’ |
| GNAT2_reverse-2 | 5’-CTCGAATTCCTACTTTGGGTACCAAAACAT-3’ |
| GNAT3_forward-2 | 5’-CATCCATGGCAACGTTGAAGAAAGACAT-3’ |
| GNAT3_reverse-2 | 5’-GTAGGATCCTTATGCCTCCAAG-3’ |
| GNAT4_forward-2 | 5’-CCATGGACGCAAGTCAAATAGTTGATCTTTTTCC-3’ |
| GNAT4_reverse-2 | 5’-GGATCCTTACCGAAACTGTTCAAGAGCTTG-3’ |
| GNAT5_forward-2 | 5’-CCATGGACACAGCTACAGAAACTGGAGAAGAAA-3’ |
| GNAT5_reverse-2 | 5’-GGATCCTTACACATTTGCAGAGGAGGTC-3’ |
| GNAT6_forward-2 | 5’-CCATGGACAGTCACTGGGAAGATCGCTCC-3’ |
| GNAT6_reverse-2 | 5’-GGATCCTTAGCTTGTGTACTGGAGCAAGTATG-3’ |
| GNAT7_forward-2 | 5’-CTGCCATGGCGTTCCGTCCCGTCGCTGCTT-3’ |
| GNAT7_reverse-2 | 5’-TCCGGTACCTTACTGGACATGATTGGGG-3’ |
| GNAT10_forward-2 | 5’-CTGCCATGGCGGCGGCGGAGATAGAGTT-3’ |
| GNAT10_reverse-2 | 5’-TCCGGTACCCTAAGAAAAGCGTTTACTC-3’ |
